# Clinical introduction of Monte Carlo treatment planning for lung stereotactic body radiotherapy

**DOI:** 10.1120/jacmp.v15i1.4202

**Published:** 2014-01-06

**Authors:** Hideharu Miura, Norihisa Masai, Ryoong–Jin Oh, Hiroya Shiomi, Kouichi Yamada, Junichi Sasaki, Toshihiko Inoue

**Affiliations:** ^1^ Miyakojima IGRT Clinic Osaka Japan

**Keywords:** Monte Carlo dose calculation, lung density, GTV size, dose prescription, stereotactic body radiation therapy

## Abstract

The purpose of this study was to investigate the impact of Monte Carlo (MC) calculations and optimized dose definitions in stereotactic body radiotherapy (SBRT) for lung cancer patients. We used a retrospective patient review and basic virtual phantom to determine dose prescriptions. Fifty‐three patients underwent SBRT. A basic virtual phantom had a gross tumor volume (GTV) of 10.0 mm with equivalent water density of 1.0 g/cm^3^, which was surrounded by equivalent lung surrounding the GTV of 0.25 g/cm^3^. D95 of the planning target volume (PTV) and D99 of the GTV were evaluated with different GTV sizes (5.0 to 30.0 mm) and different lung densities (0.05 to 0.45 g/cm^3^). Prescribed dose was defined as 95% of the PTV should receive 100% of the dose (48 Gy/4 fractions) using pencil beam (PB) calculation and recalculated using MC calculation. In the patient study, average doses to the D95 of the PTV and D99 of the GTV using the MC calculation plan were 19.9% and 10.2% lower than those by the PB calculation plan, respectively. In the phantom study, decreased doses to the D95 of the PTV and D99 of the GTV using the MC calculation plan were accompanied with changes GTV size from 30.0 to 5.0 mm, which was decreased from 8.4% to 19.6% for the PTV and from 17.4% to 27.5% for the GTV Similar results were seen with changes in lung density from 0. 45 to 0.05 g/cm^3^, with doses to the D95 of the PTV and D99 of the GTV were decreased from 12.8% to 59.0% and from 7.6% to 44.8%, respectively. The decrease in dose to the PTV with MC calculation was strongly dependent on lung density. We suggest that dose definition to the GTV for lung cancer SBRT be optimized using MC calculation. Our current clinical protocol for lung SBRT is based on a prescribed dose of 44 Gy in 4 fractions to the GTV using MC calculation.

PACS number: 87.55.D‐, 87.55.K‐

## I. INTRODUCTION

Stereotactic body radiation therapy (SBRT) plays an increasingly important role in nonsurgical treatment of early‐stage primary and secondary lung cancers. Local control during SBRT differs among institutions. Results to date indicate that a biologically effective dose (BED) larger than 100 Gy may be effective, and offers a local control rate above 90%.^1^, ^2^, ^3^, ^4^ These reports included many different dose calculation algorithms, however, and differences between planned and delivered doses were found. Dose calculation algorithms have been implemented in commercial treatment planning systems for inhomogeneous corrections sampled for scatter calculation and the inclusion or exclusion of electron transport.^(5)^ Currently the most accurate dose calculation method available, Monte Carlo (MC) calculation, models the actual physical process, leading to a dose deposition, including secondary electron distribution.^(6)^

The many studies of dosing to the target using MC calculation have found that this results in significant dose differences in clinical radiotherapy in regions with inhomogeneous materials, particularly for lung cancer.^7^, ^8^, ^9^ These differences occur because the simple algorithms such a pencil beam (PB) calculation merely account for decreased attenuation of the primary photon beam in low‐density lung tissue. Although our institution uses PB calculation for all treatments, we recently considered changing our treatment planning calculation algorithm in SBRT for lung cancer patients from PB to MC calculations. An important consideration in this decision was that MC calculation planning makes use of clinical knowledge already gained from PB calculation treatment planning.

Here, we investigated the impact of a change in calculation methods from PB to MC calculations in SBRT for lung cancer patients, and determined changes in doses delivered to patients. Our protocol for lung SBRT is 48 Gy in 4 fractions to the planning target volume (PTV) using PB calculation. We used a retrospective patient review and basic virtual phantom to determine dose prescriptions.

## II. MATERIALS AND METHODS

### A. Patient selection

Fifty‐three patients treated with SBRT between April 2009 and August 2010 were included in the analysis. PTV was 39.1±20.0 cc (7.7‐102.0 cc) and gross tumor volume (GTV) was 8.7±7.7 cc (0.5‐33.3 cc). Mean lung density was $0.23\pm 0.9\;g/{\text{cm}}^{3}$ (0.13‐0.36 g/cm^3^). Patients with tumors near the esophagus and bronchus were excluded.

### B. Treatment planning

The 53 patients underwent four‐dimensional 4D CT (four‐slice BrightSpeed QX/i scanner; General Electric Medical Systems, Milwaukee, WI) to more accurately determine tumor shape, volume, and position at different phases of the breathing cycle. Slice thickness was 2.5 mm and gantry rotation time was 1.0 second. Each image was tagged with the corresponding phase of the respiratory cycle and then sent to a GE Advantage 4.0 workstation (General Electric) using the Advantage 4D CT software. The 4D datasets were categorized into the four respiratory cycle phases of 0%, 25%, 50%, and 75%. The visible tumor was delineated as the GTV in the CT pulmonary window by the oncologist. Internal target volume (ITV) was the sum of amplitudes of the GTV. No additional margin was added for generation of the clinical target volume (CTV). The PTV was generated by adding a uniform margin of 8 mm to the ITV to account for setup uncertainties and mechanical accuracy. Treatment fields were conformed around the PTV. A leaf margin of 2 mm was added to the PTV, and the isocenter was positioned in the center of the PTV. The plan was calculated using PB calculation in the iPlan RT Dose ver. 4.1.2 treatment planning system (BrainLAB, Munich, Germany). Beam energy for all plans was 6 MV photons. Dose prescription was defined such that 100% of the prescribed dose covered 95% of the PTV. The prescription dose was 48 Gy in 4 fractions. The irradiated lung volume was made as small as possible. Beam arrangement used noncoplanar and nonopposing beams. The plan was then recalculated using MC calculation while maintaining the same planning parameters, namely for beam arrangement, leaf positions, isocenter, position, and monitor unit, using the full MLC geometry simulation ‘Accuracy Optimized Model’ with a spatial resolution of 2 mm and variance of 2%.

### C. Phantom study

**Figure 1:**
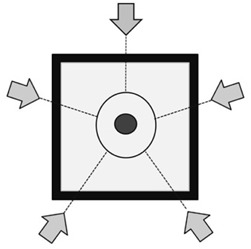
Schematic overview of the foundation model with the center of the 10 mm spherical tumor (black) surrounded by lung‐equivalent material (grey) and a 20 mm wall of water‐equivalent material. Directions of the five beams are indicated by arrows. Margin for the PTV was 8.0 mm in all directions.

Figure 1 shows the basic virtual phantom, which had a simulated GTV of 10 mm diameter and an equivalent water density of 1.0 g/cm^3^. Density of the equivalent lung surrounding the GTV was defined as 0.25 g/cm^3^. GTV diameter was assigned a series of sizes of 5.0, 10.0, 15.0, 20.0, 25.0, and 30.0 mm with a fixed lung density of $0.25\;g\;{\text{cm}}^{-3}$. Lung density was 0.05, 0.15, 0.25, 0.35, and 0.45 g/cm^3^ with a fixed GTV diameter of 10 mm. The selected GTV sizes were intended to represent average target dimensions in lung SBRT, and lung density was selected to cover the normal range of lung density. Gantry angles were 0°, 72°, 144°, 216°, and 288°, which were consistent with simple SBRT treatment planning. Beam energy for phantom study was 6 MV photons.

### D. Plan analyses

This study evaluated shape differences in the dose‐volume histograms (DVH) for the different calculations using the same plan. For each treatment plan, we evaluated the minimum dose to 95% of the PTV (D95), minimum dose to 99% of the GTV (D99), and lung volume receiving 5, 10, and 20 Gy or more (V5, V10, and V20, respectively), together with the mean dose. Data were analyzed using the two‐sided paired Student's t‐test with statistical significance set at p<0.05. Statistical analysis was performed with the Statistical Package of Social Sciences, v.19.0 (IBM, Chicago, IL)

## III. RESULTS

**Figure 2:**
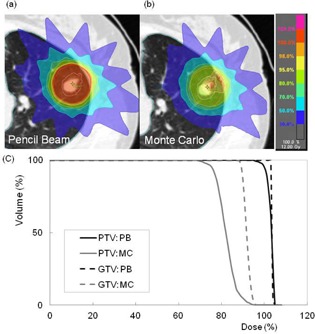
Dose distributions calculated using (a) PB and (b) MC calculations for a patient with lung cancer; (c) comparison of dose‐volume histograms between PB (black line) and MC (gray line) calculation plans for the same patient. Compared to PB, MC calculation plans provided substantially lower doses to the target.

**Figure 3:**
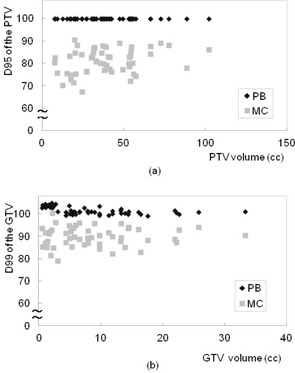
Plots of (a) D95 of the PTV and (b) D99 of the GTV as a function of target size using PB (black) and MC (gray) calculation plans in the patient study. D95 of the PTV and D99 of the GTV using the MC calculation plan were on average 19.9% and 10.2% lower than those with the PB calculation plan, respectively.

**Figure 4:**
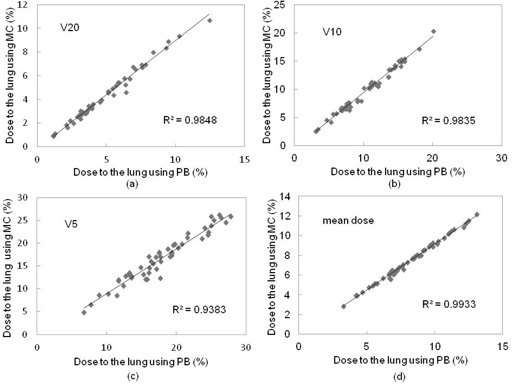
Plots of (a) V20, (b) V10, (c) V5, and (d) mean dose to the lung using PB and MC calculation plans in the patient study. V5, V10, V20, and mean doses to the lung using the MC calculation plan were on average 6.7%, 6.8%, 12.5%, and 8.6% lower than those with the PB calculation plan, respectively.

Figure 2 shows representative axial dose distributions and DVH of PB and MC calculations for the same patient. Dose distribution was significantly changed with MC calculation, and led to a decrease in dose to the target. Figures 3 and 4 show the plot of doses to the target and lung using PB and MC calculations. Table 1 compares the PB and MC calculation plans with regard to D95 of the PTV, D99 of the GTV, and doses to the lung. D95 of the PTV and D99 of the GTV using the MC calculation plan were on average 19.9% and 10.2% lower than those with the PB calculation plan, respectively. Further, the V5, V10, V20, and mean doses to the lung using the MC calculation plan were on average 6.7%, 6.8%, 12.5%, and 8.6% lower than those with the PB calculation plan, respectively. The dose to the lung using the MC calculation plan was linearly related to that using the PB plan with a coefficient of determination $\left(R^{2}=0.94-0.99\right)$. Statistically significant differences were seen in all evaluation parameters (p<0.001).

**Table 1:** Dosimetric comparisons between PB and MC calculation plans, with data shown as group averages and standard deviation with ranges in parentheses (n=53). The two‐sided paired Student's *t*‐test showed a statistically significant difference for all evaluation parameters (p<0.001)

		*PB*	*MC*
PTV	D95 (%)	100±0.0 (100.0−100.0)	80.1±5.5 (67.4–90.5)
GTV	D99 (%)	101.6±1.6 (99.2–105.0)	89.8±4.3 (79.0–100.3)
Lung	V20 (%)	4.8±2.4 (1.1–12.5)	4.2±2.2 (0.9–10.7)
	V10 (%)	10.3±4.0 (3.1–20.1)	9.6±4.0 (2.5–20.3)
	V5 (%)	17.9±5.2 (6.8–27.8)	16.7±5.3 (4.9–26.2)
	Mean Dose (%)	8.1±2.3 (3.3–13.1)	7.4±2.1 (2.8–12.2)

**Figure 5:**
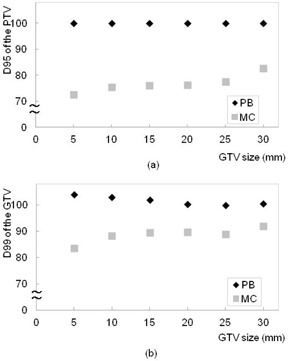
Plots of (a) D95 of the PTV and (b) D99 of the GTV as a function of GTV size using PB (black) and MC (gray) calculation plans in the phantom study. Dose to the PTV using MC calculation gradually decreased with decreasing GTV size.

**Figure 6:**
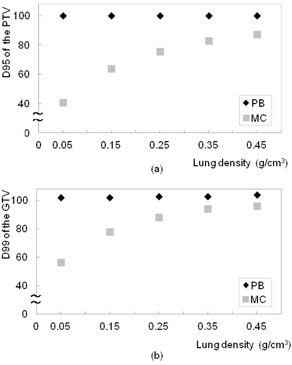
Plots of (a) D95 of the PTV and (b) D99 of the GTV as a function of lung density using the PB (black) and MC (gray) calculation plans in the phantom study. Dose to the PTV using MC calculation gradually decreased with decreasing lung density.

For the basic phantom condition (GTV size 10 mm, and lung density 0.25 g/cm^3^), doses to the 95% of the PTV and 99% of the GTV using the MC calculation plan were 24.5% and 11.7% lower than those with the PB plan, respectively. Figure 5 shows the plot of dose to the 95% of the PTV and 99% of the GTV as a function of GTV size between the PB and MC calculation plans. Of the compared GTV sizes (30.0 to 5.0 mm), the decrease in dose to D95 of the PTV and D99 of the GTV using the MC calculation plan ranged from 17.4% to 27.5% (difference: 10.1%) and 8.4% to 19.6% (difference: 11.2%), respectively. Figure 6 shows the plot of dose to the PTV and GTV as a function of lung density between the PB and MC calculations. Of the compared lung densities (0.45 to 0.05 g/cm^3^), the decrease in dose to D95 of the PTV and D99 of the GTV using the MC calculation plan ranged from 12.8% to 59.0% (difference: 46.2%) and 7.6% to 44.8% (difference: 37.2%), respectively.

## IV. DISCUSSION

Dose prescription in SBRT is defined by isocenter or volume prescription methods. The Japan Clinical Oncology Group (JCOG) and Radiation Therapy Oncology Group (RTOG) have planned multi‐institutional trials of SBRT for non‐small cell lung cancer. In the JCOG 0403 protocol, dose prescription is defined as the point dose at the isocenter of the PTV with inhomogeneous correction, such as the pencil beam convolution with Batho power‐law and Clarkson with effective path length correction, but this prescription is not accurate for dose calculations of lung cancer.^(10)^ In contrast, the RTOG 0236 protocol defines dose prescription as the volume dose at the periphery of the PTV without inhomogeneous correction.^(11)^ The dose maximum within the PTV should preferably not be less than 110% or exceed 140% of the prescribed dose, similar to the criteria formulated in RTOG protocol 0618.^(12)^ Therefore, comparing the actual delivered doses among these trials is difficult. Dose differences between the planned and actually delivered radiation doses may impact the probability of local tumor control.

PB calculation was performed by applying an equivalent path length (EPL) correction without considering variation in lateral electron disequilibrium. This results in the underestimation of penumbra width in low‐density materials and overestimation of dose to the target.^(13)^ In contrast, MC calculation can be considered to provide transport accuracy under conditions of electronic disequilibrium.^(14)^ In this study, we found that MC and PB calculations gave significantly different results for GTV and PTV and dose to the lung; D95 of the PTV and D99 of the GTV with MC calculation were on average 19.9% and 10.2% lower than those with the PB calculation plan, respectively. These findings suggest the need for particular attention when changing dose calculation algorithms for lung cancer patients. Large variations were observed between individual patients, however, possibly due to differences in GTV volume, lung density, and tumor location.^(15)^ Although we considered the relationships between these factors and differences, it was unclear to which location and size the differences were significant.

Many researchers have reported the conversion of prescription dose from PB to MC calculations for lung cancer treatment planning.^15^, ^16^, ^17^, ^18^ Some examples include the follow. A PTV dose of 3×20 Gy with the EPL calculation was equivalent to a dose of 3×18 Gy with inhomogeneous correction calculation.^(16)^ In CyberKnife treatment, a current dose of 3×20 Gy was converted to the following dose schedules: 3×16 Gy for tumors <3 cm, 3×17 Gy for tumors of 3‐5 cm, and 3×18 Gy for tumors >5 cm.^(15)^ These reported dose prescriptions were defined at the PTV. RTOG 0236 protocol in terms of the total prescribed dose and dose fractionation uses a prescribed dose of 18–19 Gy for three treatments instead of the 60 Gy used for homogeneous tissue density calculations.^(17)^ Narabayashi et al.^(18)^ reported that the Batho power‐law correction plan (BPL) was close to the MC calculation plan, and switched the heterogeneity correction algorithm from BPL to MC calculations under isocenter prescription because the differences in monitor units between BPL and MC calculations were small (−1.4%). However, the International Commission on Radiation Units and Measurements (ICRU) recommended the methods of dose prescription have changed from prescription at the isocenter point of the treatment plan to prescription dose at the periphery of the PTV.^(19)^ In Japan, D95 prescription was adopted in the JCOG 0702 phase I trial instead of the isocenter prescription adopted in the previous JCOG 0403 phase II trial.

**Figure 7:**
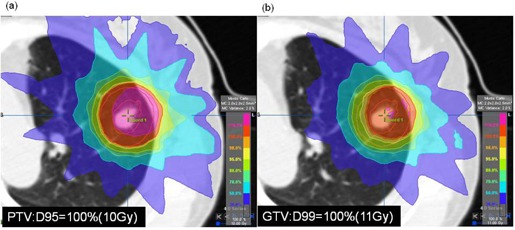
Dose prescription was defined at (a) PTV and (b) GTV for lung SBRT.

Based on the average dose reduction in target, we have shown that a D95 to the PTV of 48 Gy using the PB calculation plan was equivalent to a D95 to the PTV of 40 Gy and a D99 of the GTV of 44 Gy using the MC calculation plan. Which is better — PTV or GTV on prescription dose? Our phantom study suggested that dose to the PTV was strongly dependent on GTV size and lung density,^(20)^ but that these variables had less effect on dose to the GTV. Dose calculation to the PTV is complicated, however, because it can be affected by low lung density. If the dose prescription is defined at the PTV, monitor units should be adjusted to allow sufficient PTV coverage (Fig. 7). The external border of the PTV is covered by a lower isodose surface than that usually used in conventional radiotherapy planning, typically around 80%.^21^, ^22^ Decreased dose to the tumor may be effective on tumor local control probability, especially when the lung density is decreased. The PTV is a tool designed to ensure that the CTV receives an adequate absorbed dose.^(19)^ Additionally, our previous study supported the clinical acceptability of treatment planning for breathing‐induced tumor motion based on the dose prescription defined to the GTV.^(23)^ Dose prescription to the GTV is accordingly more highly optimized than that to the PTV. We intend to perform a prospective analysis to define 99% of the GTV receiving a minimum of 100% of the dose using an MC calculation for lung SBRT. Given that dose differences generally increase with decreasing target size,^(15)^ additional clinically‐based discussion of changes in dose by tumor volume and metastasis might be valuable.^1^, ^2^, ^3^, ^4^, ^24^ Further research is aimed to define a new dose effect determining the true tumor control probability and normal tissue complication probability using MC calculation.

## V. CONCLUSIONS

In this study, we found that doses calculated using an MC calculation plan provided a dose decrease in target coverage compared to those calculated with a PB calculation plan. Our phantom study suggested that dose to the PTV was strongly dependent on GTV size and lung density. Our current clinical protocol for lung SBRT is based on a prescribed dose of 44 Gy in 4 fractions to the GTV using MC calculation, instead of 48 Gy in 4 fractions to the PTV using PB calculation. Our goal is to optimize high‐dose precision radiation therapy for lung SBRT using MC calculation.

## Supporting information

Supplementary Material

Supplementary Material
