# Supplementary figures and images for: Clinical introduction of Monte Carlo treatment planning for lung stereotactic body radiotherapy
Source: J Appl Clin Med Phys. 2014 Jan 6;15(1):38–46. doi: 10.1120/jacmp.v15i1.4202 (PMC5711232; doi:10.1120/jacmp.v15i1.4202)

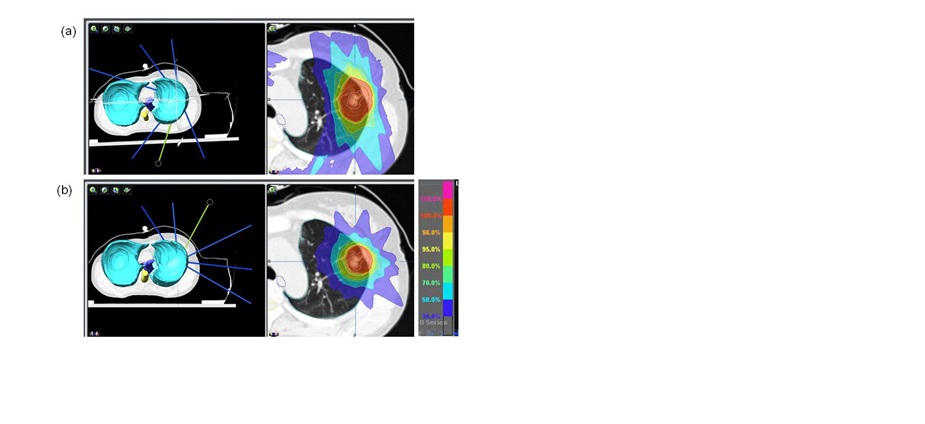

Supplement: Supplementary file 1 — Supplementary Material [file ACM2-15-038-s001.jpg]

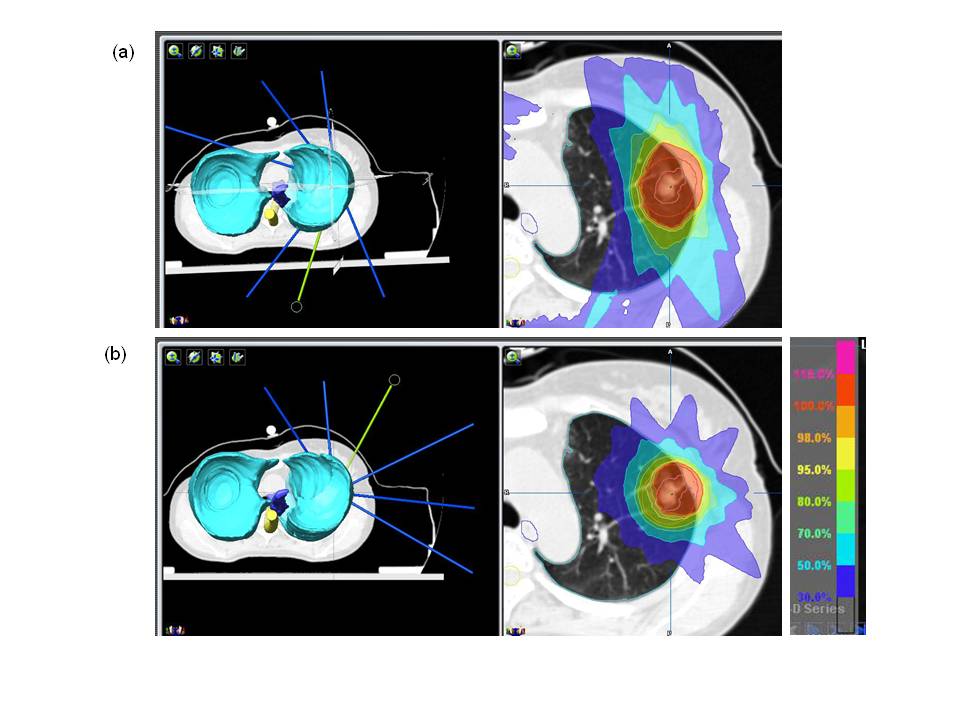

Supplement: Supplementary file 2 — Supplementary Material [file ACM2-15-038-s002.JPG]
